# Supplementary material for: Modeling longitudinal imaging biomarkers with parametric Bayesian multi‐task learning
Source: Hum Brain Mapp. 2019 Jun 5;40(13):3982–4000. doi: 10.1002/hbm.24682 (PMC6679792; doi:10.1002/hbm.24682)
Supplement: Supplementary file 1 — Figure S1 Left: histogram of intercept estimates from a representative MTL model (“plain”) in red versus ordinary least squares' estimates in blue, with corresponding red and blue dashed lines indicating each model's mean estimates and black line indicating the true mean across intercepts. Right: same for slope estimates. Figure S2 Boxplots of models' prediction coverage probabilities for intercept variation (top figure) and slope variation (bottom figure) simulations. Figure S3 Simulations comparing proposed empirical Bayesian realization of “plain” (“EB plain”) to its full Bayesian realization via MCMC sampling (“MCMC plain”). A: boxplots of prediction errors (log10MAE) and prediction coverage probabilities for both models. Top row is intercept varying scenario, bottom row is slope varying scenario. B: corresponding boxplots of parameter coverage and parameter prediction error for both scenarios. Figure S4 A: boxplots of prediction errors (log10MAE) and prediction coverage probabilities for simulations varying measurement noise correlation (parameter ρ) for four representative models. Top row is intercept varying scenario, bottom row is slope varying scenario. B: corresponding boxplots of parameter coverage and parameter prediction error for both scenarios. Figure S5 A: depiction of Gaussian distributed measurement noise (zero skewness) and two skewed distributions. B: boxplots of prediction errors (log10MAE) and prediction coverage probabilities for simulations these three levels of error skewness for four representative models. Top row is intercept varying scenario, bottom row is slope varying scenario. C: corresponding boxplots of parameter coverage and parameter prediction error for both scenarios. Figure S6 Histograms of residuals for the “CSF tau/aBeta” (blue) and “OLS” (rose) models for each region of interest in the ADNI application. Figure S7 Boxplots (plus mean value as circle) of absolute errors between actual and predicted annualized rate of change fro [file HBM-40-3982-s001.docx]

# **Supplementary Material**


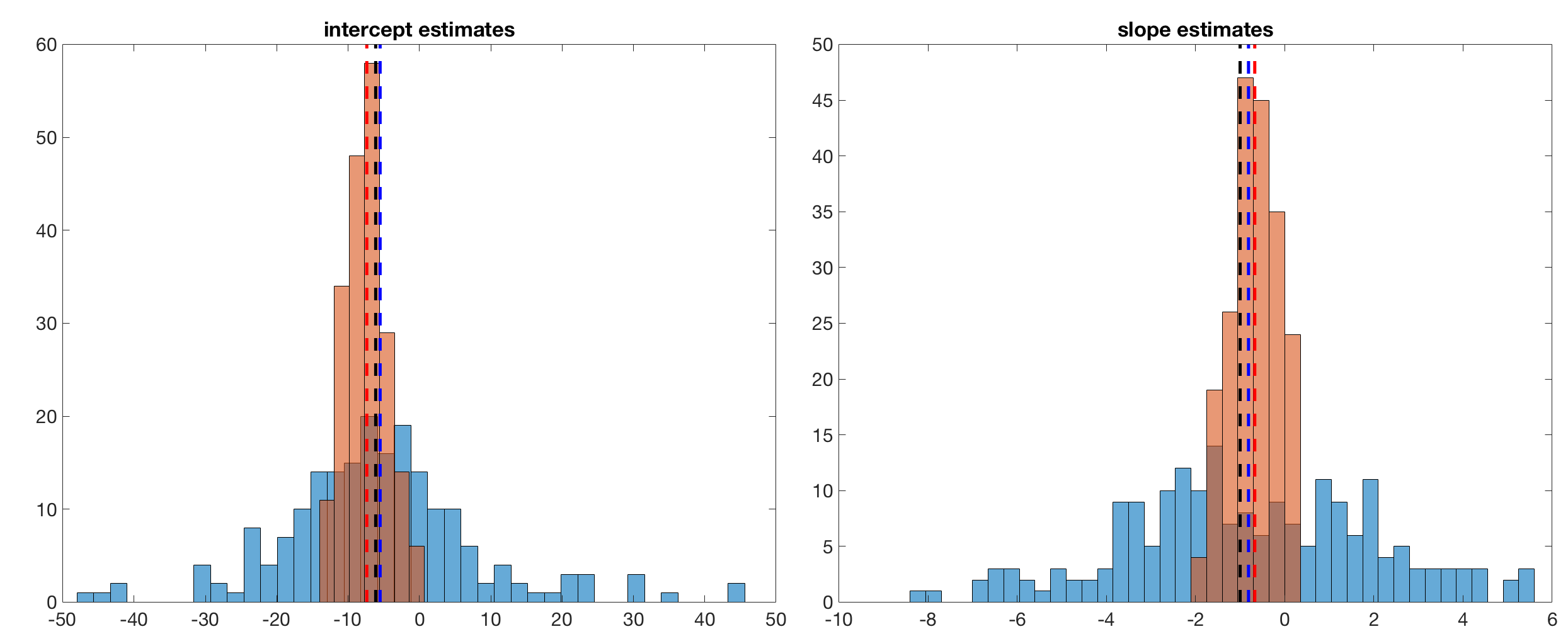


**Figure S1** **Left:** histogram of intercept estimates from a representative MTL model (‘*plain*’) in red versus ordinary least squares’ estimates in blue, with corresponding red and blue dashed lines indicating each model’s mean estimates and black line indicating the true mean across intercepts. **Right:** same for slope estimates.

**
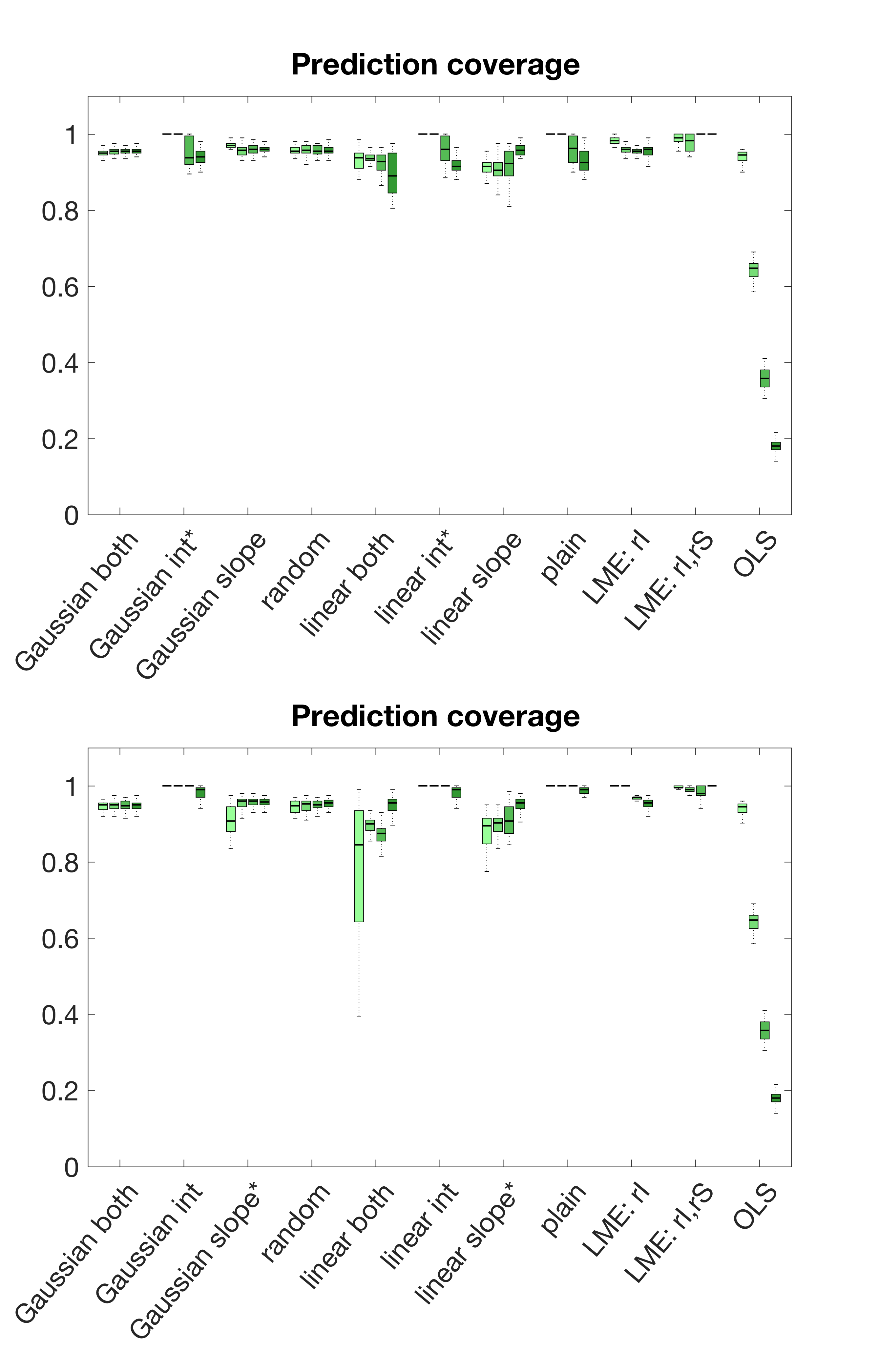
**

**Figure S2** Boxplots of models’ prediction coverage probabilities for intercept variation (top figure) and slope variation (bottom figure) simulations.

*
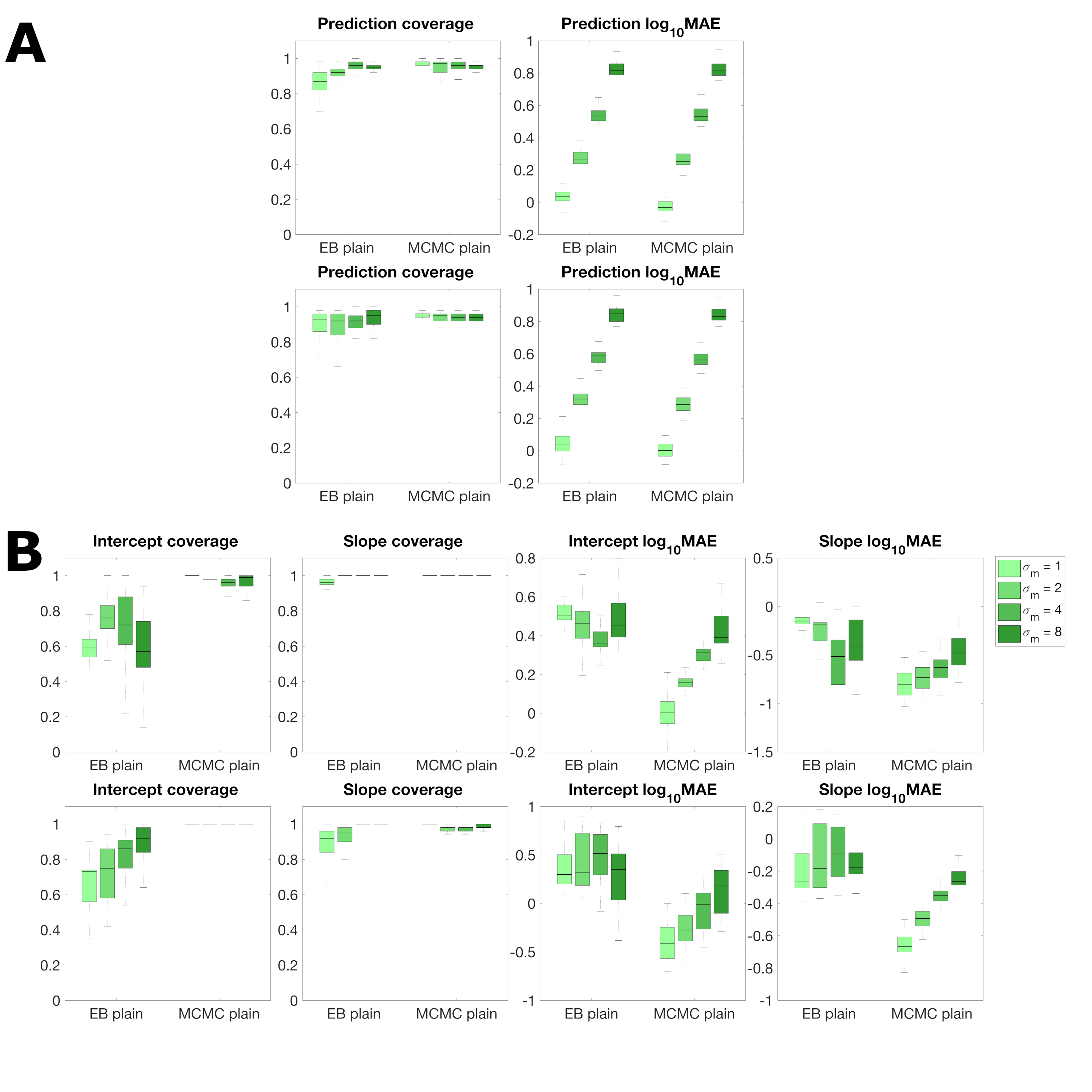
*

**Figure S3** Simulations comparing proposed empirical Bayesian realization of ‘*plain*’ (‘*EB plain*’) to its full Bayesian realization via MCMC sampling (‘*MCMC plain*’). **A:** boxplots of prediction errors (log_10_MAE) and prediction coverage probabilities for both models. Top row is intercept varying scenario, bottom row is slope varying scenario. **B:** corresponding boxplots of parameter coverage and parameter prediction error for both scenarios.

***
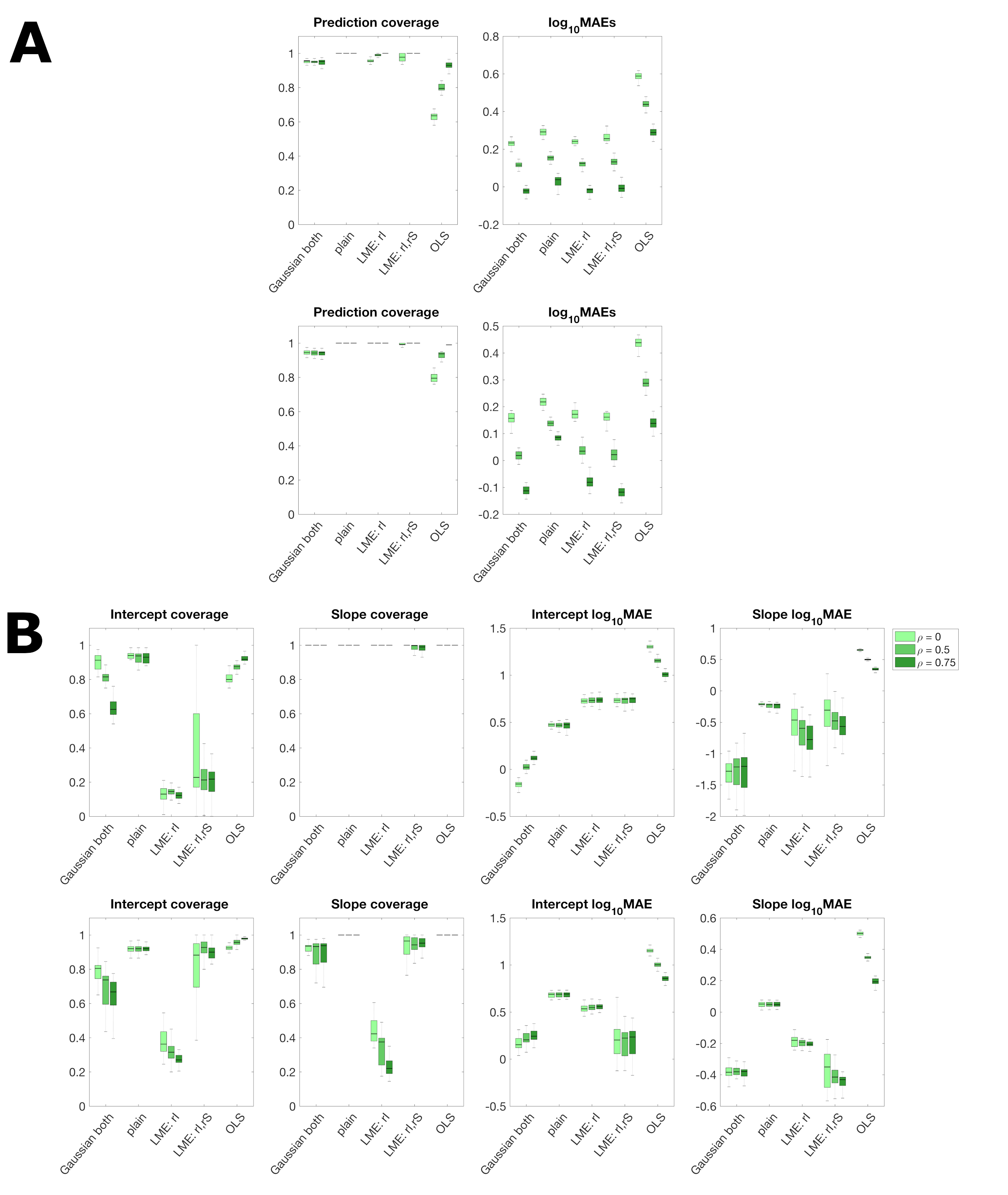
***

**Figure S4** **A:** boxplots of prediction errors (log_10_MAE) and prediction coverage probabilities for simulations varying measurement noise correlation (parameter $\rho$) for four representative models. Top row is intercept varying scenario, bottom row is slope varying scenario. **B:** corresponding boxplots of parameter coverage and parameter prediction error for both scenarios.

**
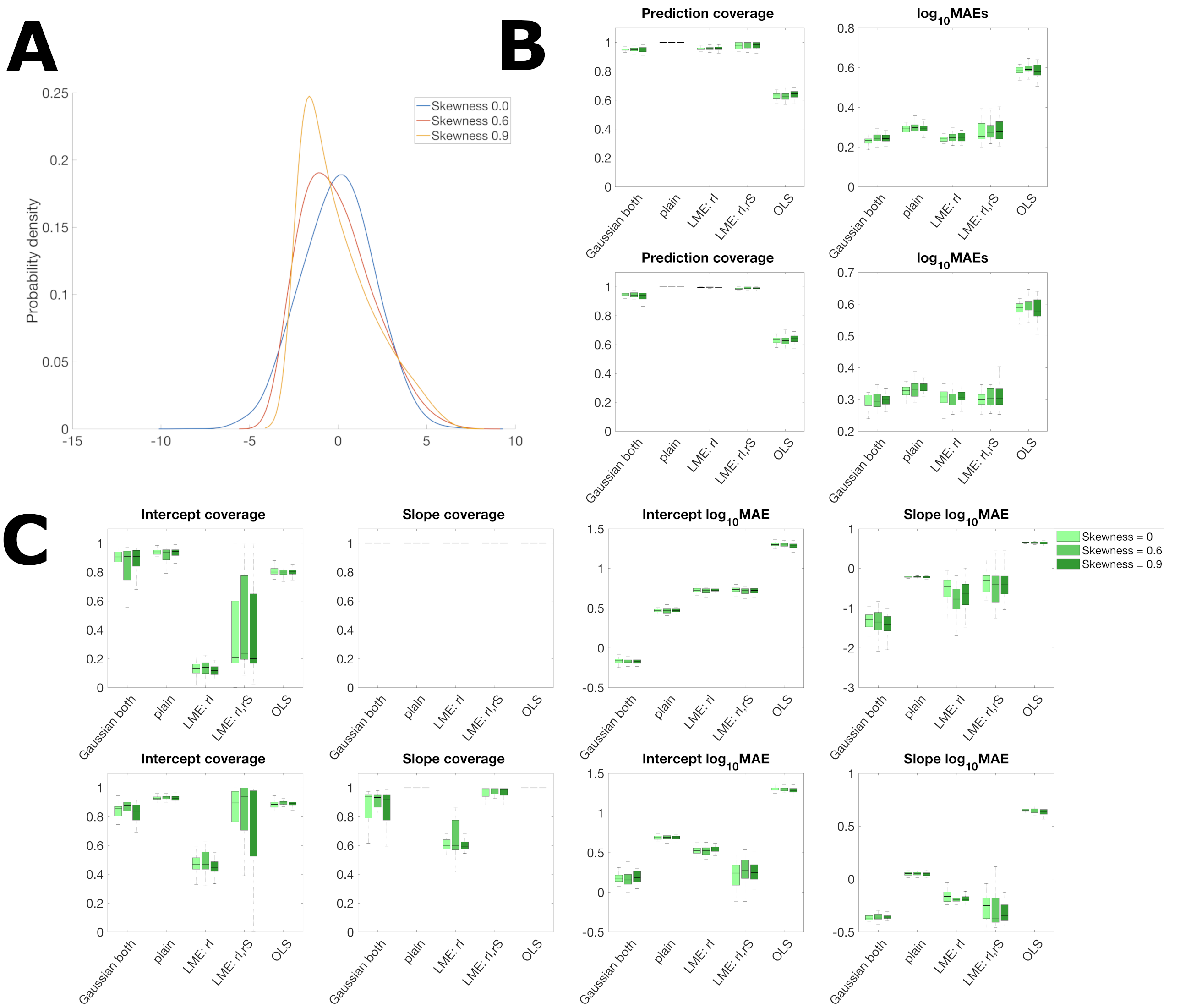
**

**Figure S5** **A:** depiction of Gaussian distributed measurement noise (zero skewness) and two skewed distributions. **B:** boxplots of prediction errors (log_10_MAE) and prediction coverage probabilities for simulations these three levels of error skewness for four representative models. Top row is intercept varying scenario, bottom row is slope varying scenario. **C:** corresponding boxplots of parameter coverage and parameter prediction error for both scenarios.


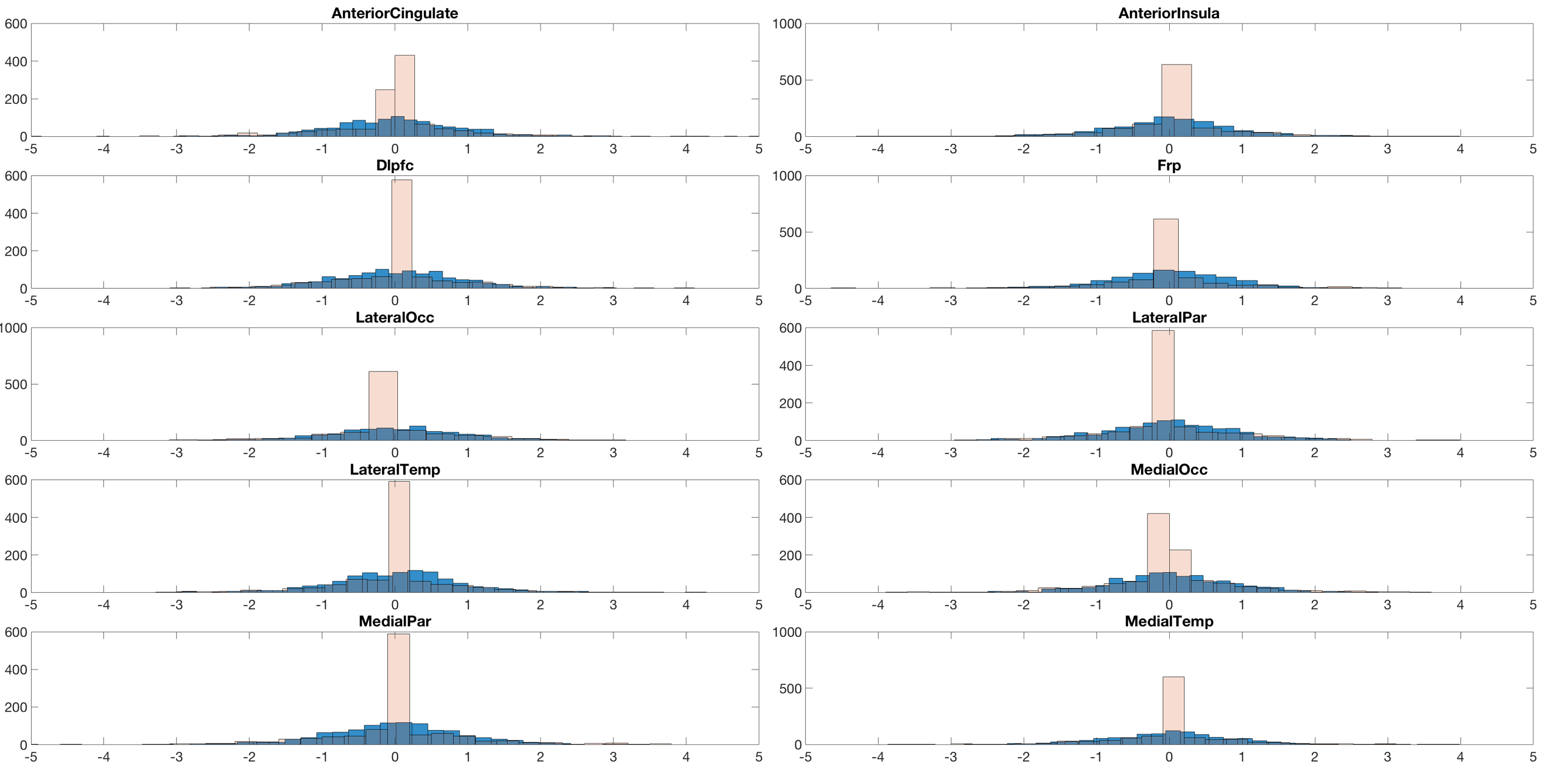


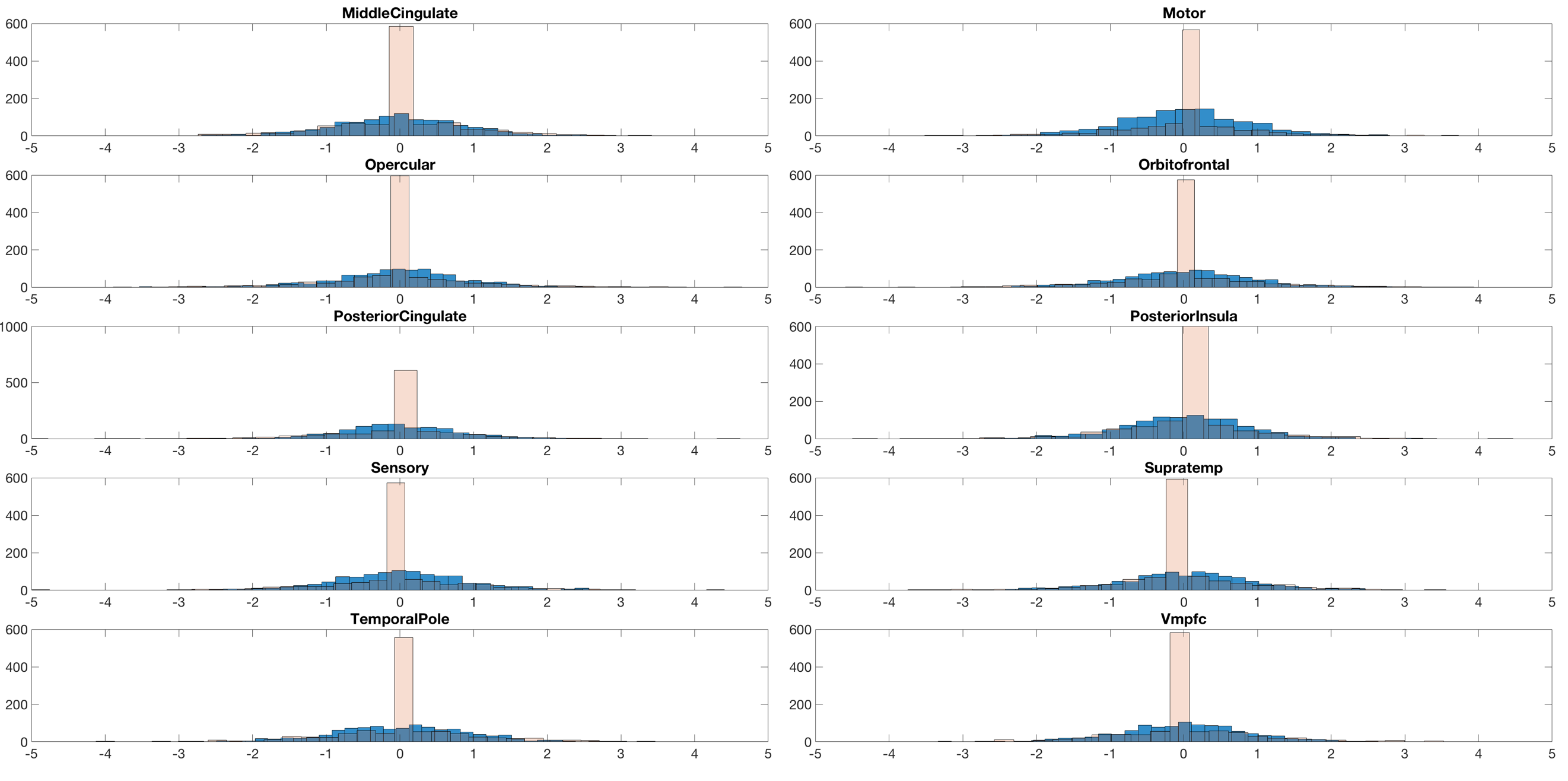


**Figure S6** Histograms of residuals for the ‘*CSF tau/aBeta*’ (blue) and ‘*OLS*’ (rose) models for each region of interest in the ADNI application.

**
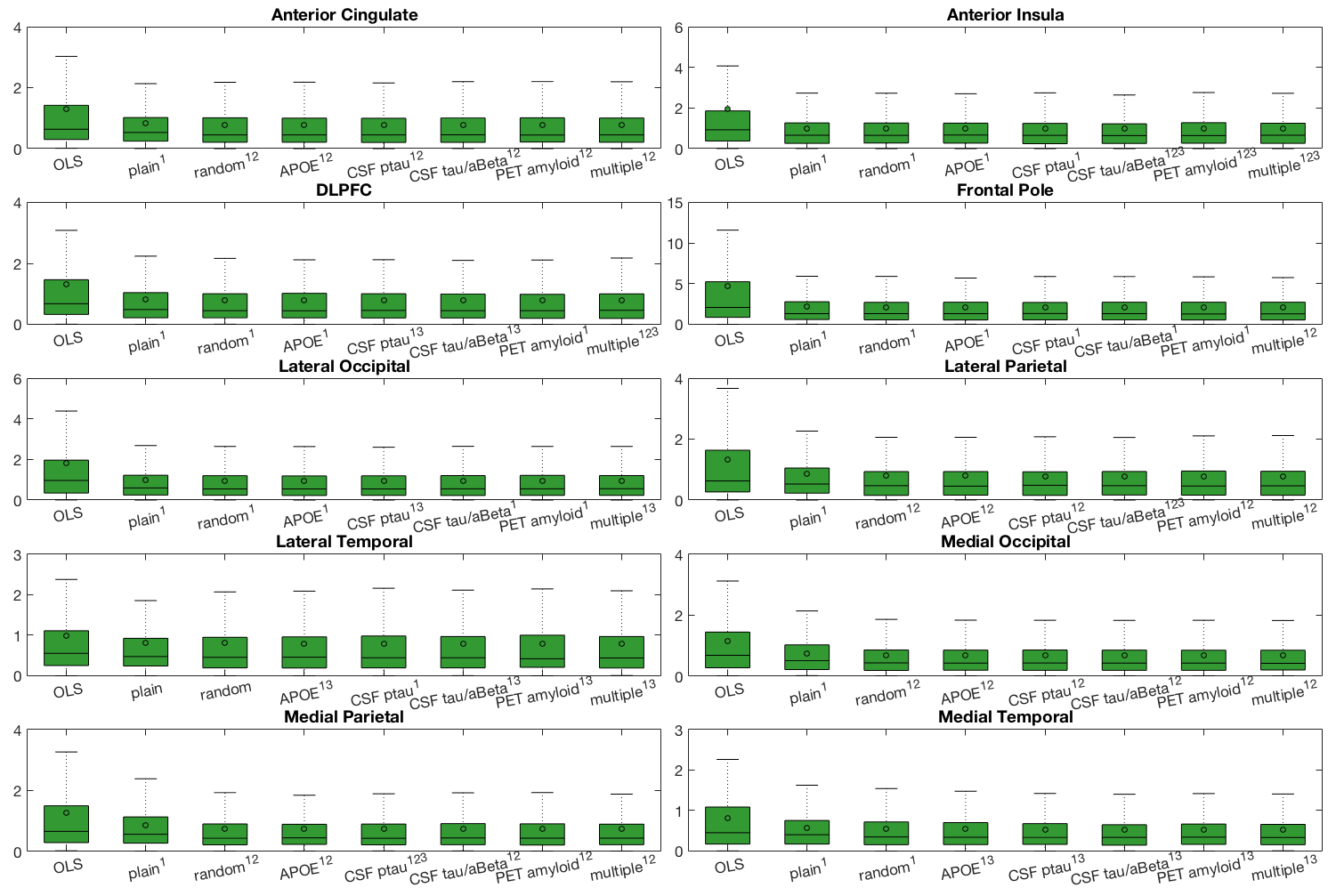

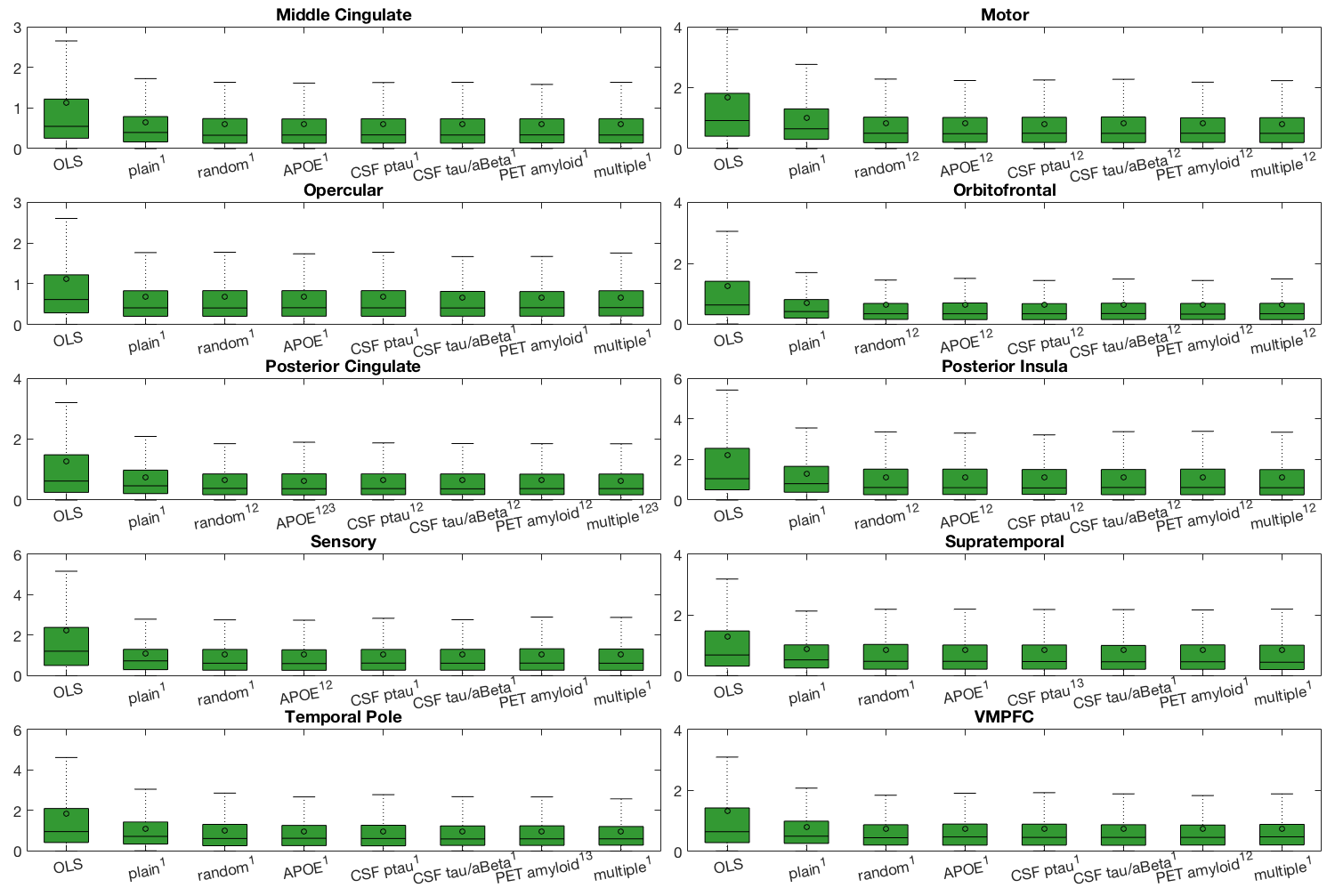
 Figure S7** Boxplots (plus mean value as circle) of absolute errors between actual and predicted annualized rate of change from baseline to final (out-of-sample) follow-up (y-axis), across all models (x-axis) and cortical ROIs (panels). Superscripts: significantly lower MAE (p < 0.05) of given model compared to 1: ‘OLS’; 2: ‘plain’; 3: ‘random’. Abbreviations: DLPFC: dorsolateral prefrontal cortex; VMPFC: ventromedial prefrontal cortex.


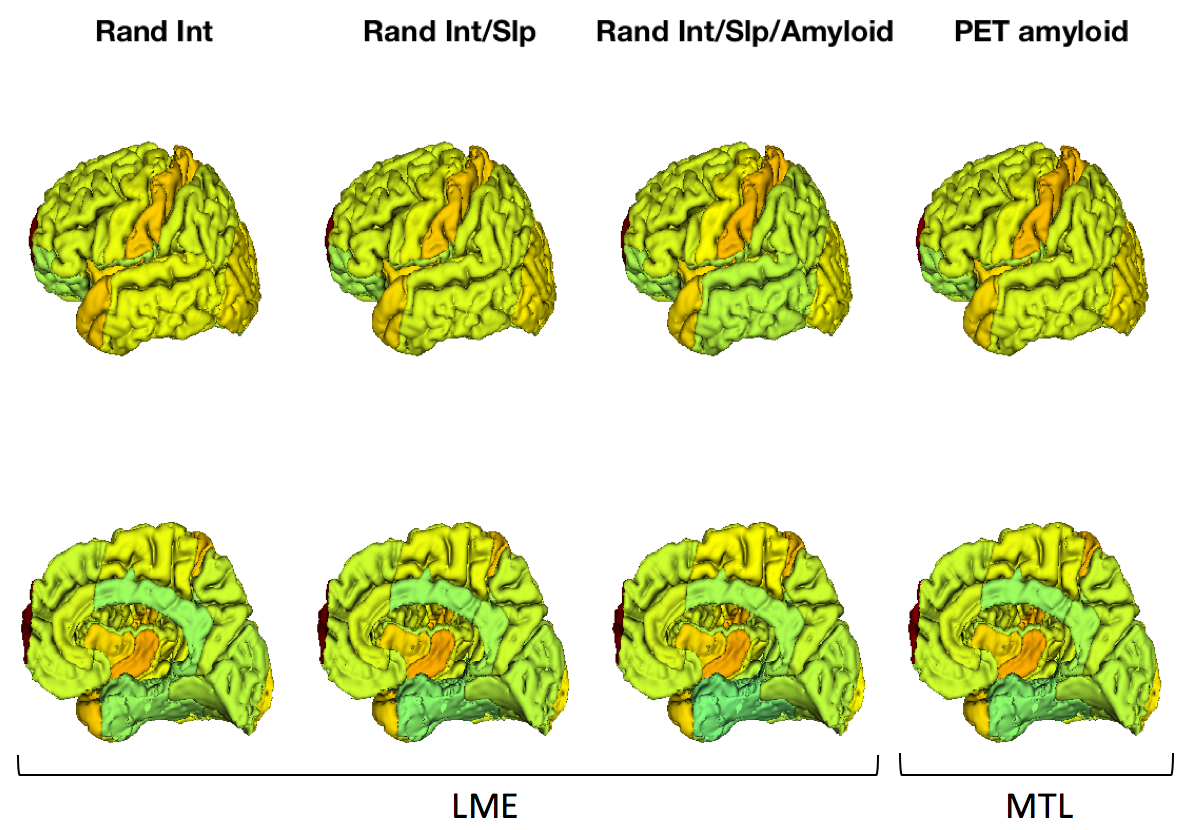


**Figure S8** Prediction errors (MAEs of annualized rates of change) for three LME models and most comparable MTL model (‘*PET amyloid*’).


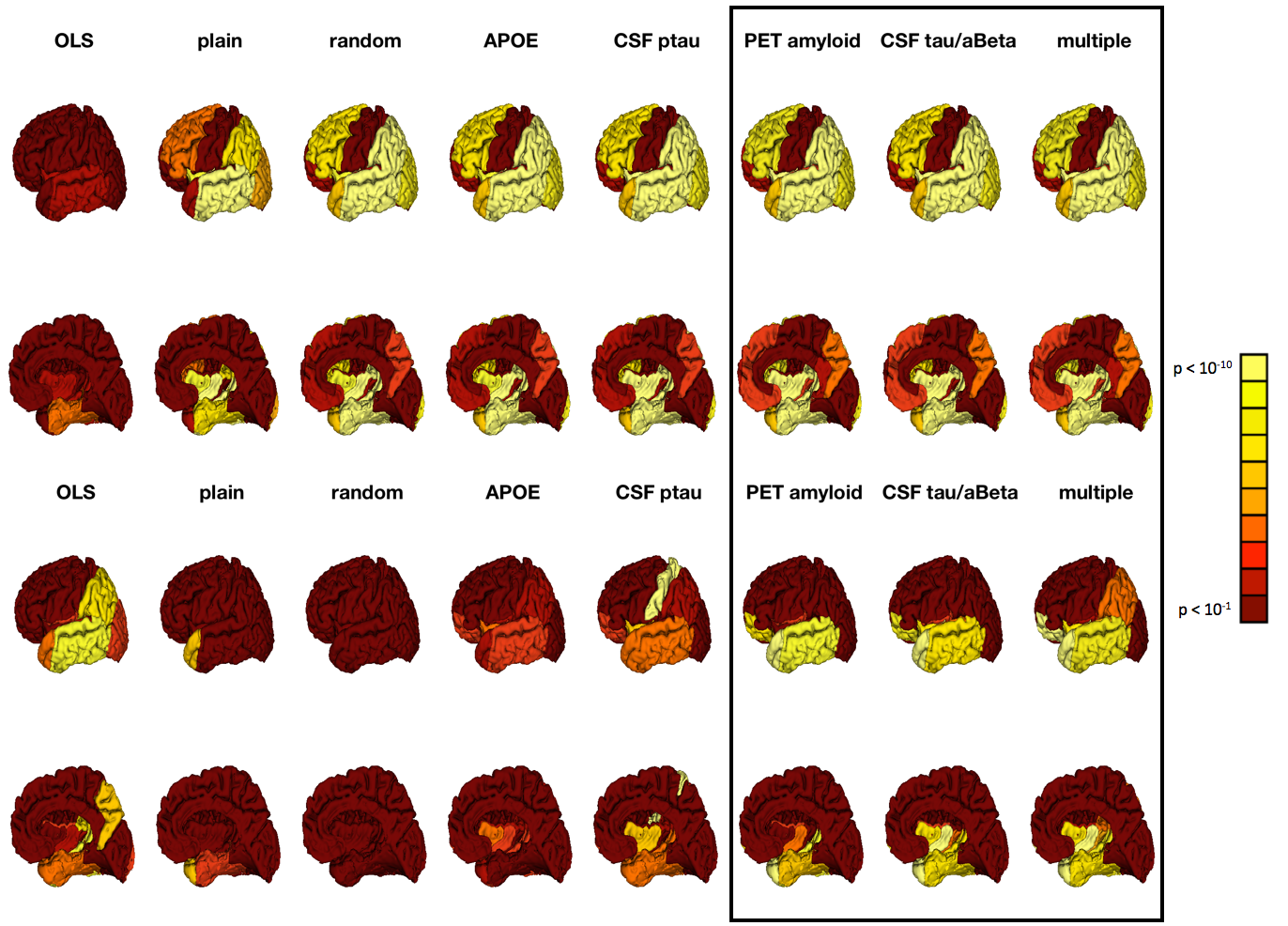


**Figure S9 Top:** Significance of (cross-sectional) diagnostic group differences in predicted volume at mean baseline age (73.5 years) across cortex for all MTL models **Bottom:** Same for (longitudinal) group differences in estimated slopes across all MTL models. ‘CSF tau/aBeta’, ‘PET amyloid’, ‘multiple’ have largest model evidence relative to ‘random’ (see Figure 5).


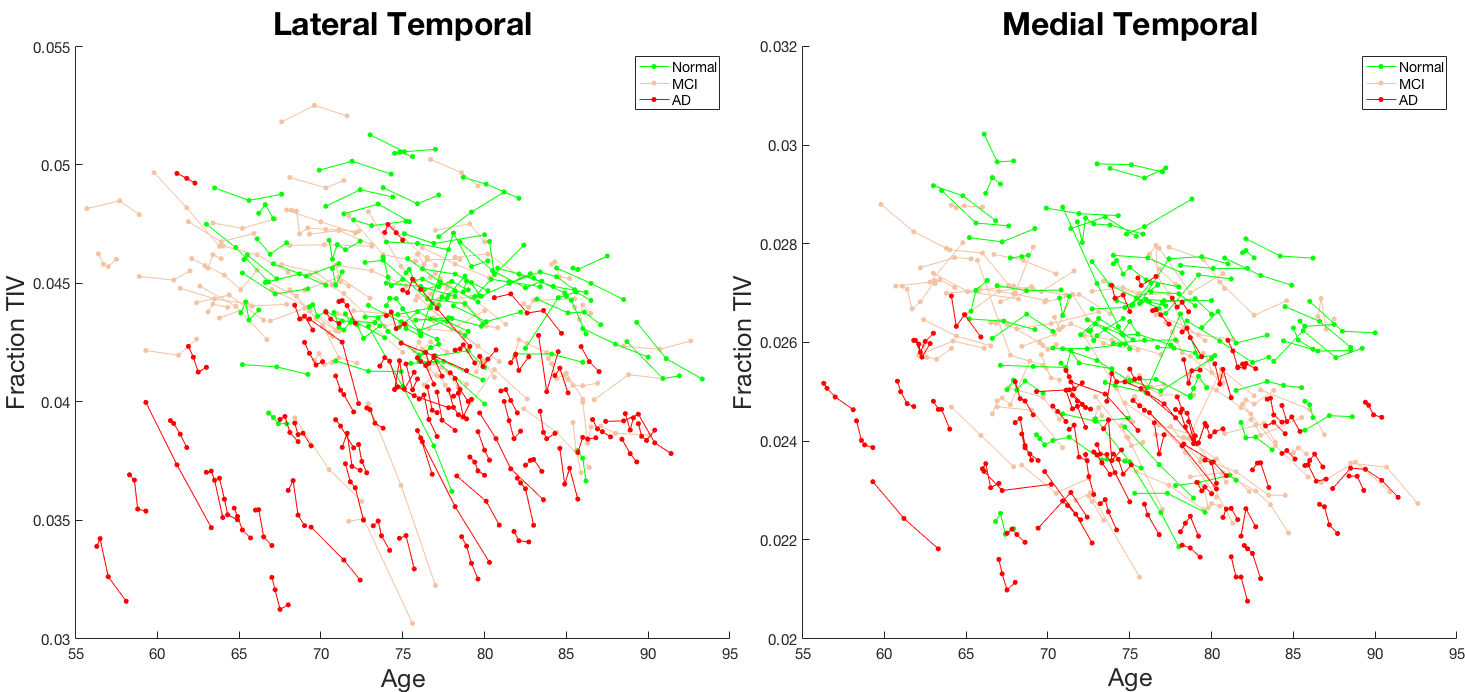


**Figure S10** All available data-points for seventy randomly selected subjects per group in two regions (210 subjects total), showing both (cross-sectional) group differences at mean age (73.5 years) and (longitudinal) differences in rates of change.


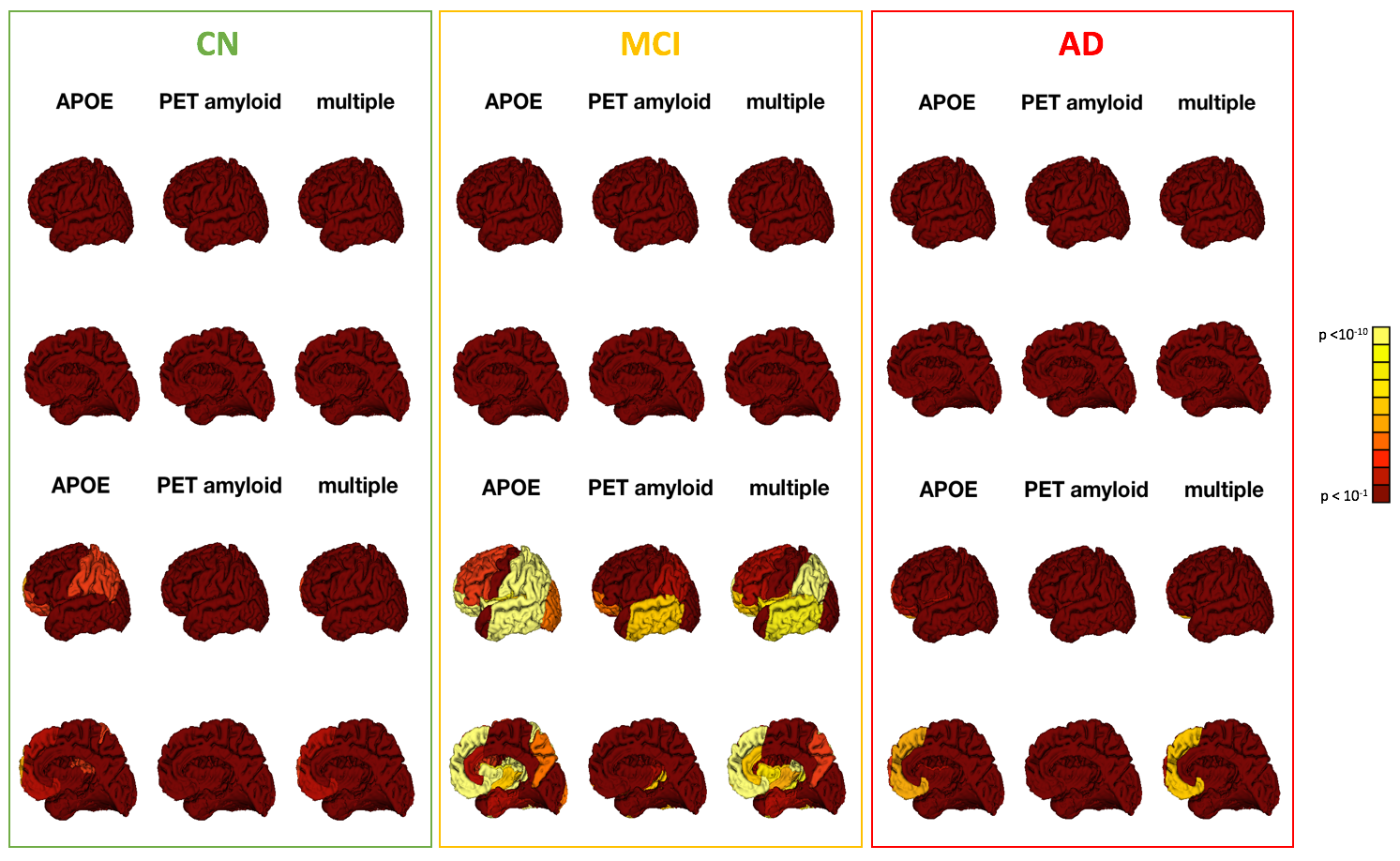


**Figure S11 Top:** Effect of the number of APOE ε4 alleles on cortical volume at mean baseline age (73.5 years) within each diagnostic group for three representative models, Bonferroni corrected for all comparisons **Bottom:** Same for effect of number of alleles on estimated slopes.

**Table S1** Convergence diagnostics for the hyperparameters of the ‘*MCMC plain*’ model for one representative run of the intercept varying simulation scenario. MCSE is the Markov Chain standard error, defined as the standard deviation ($\sigma$) divided by $\sqrt{N_{\mathrm{eff}}}$, where $N_{\mathrm{eff}}$ is effective sample size.

| **Name** | $\boldsymbol{\mu}$ | $\boldsymbol{\sigma}$ | **N_eff_** | $\hat{\mathbf{R}}$ | **MCSE** | **Confidence Interval** |
| --- | --- | --- | --- | --- | --- | --- |
| $\alpha_{1}$ | 0.032 | 0.032 | 479 | 1 | 0.0014 | (0.029, 0.035) |
| $\alpha_{11}$ | 4.9 | 1.4 | 4000 | 1 | 0.022 | (4.85, 4.94) |
| $\alpha_{12}$ | 75 | 56 | 4000 | 1 | 0.89 | (73.3, 76.7) |
| $\alpha_{21}$ | 0.033 | 0.033 | 459 | 1 | 0.0015 | (0.030, 0.036) |
| $\alpha_{22}$ | 55 | 52 | 4000 | 1 | 0.83 | (53.4, 56.6) |
| $\beta^{-1}$ | 1.2 | 0.24 | 1539 | 1 | 0.0062 | (1.19, 1.21) |
